# Normal and slow learners: a new discriminative method based on the speed of spatial learning in aged mice

**DOI:** 10.3389/fnagi.2025.1567929

**Published:** 2025-06-06

**Authors:** Céline Duffau, Senka Hadzibegovic, Vojislav Andelkovic, Bruno Bontempi, Olivier Nicole

**Affiliations:** ^1^Faculty of Science, Brigham Young University - Hawaii, Laie, HI, United States; ^2^INCIA, CNRS UMR 5287, Bordeaux, France; ^3^University of Bordeaux, Bordeaux, France; ^4^IINS, CNRS UMR 5297, Bordeaux, France

**Keywords:** aging, individual difference, normal aging, radial arm maze, spatial memory

## Abstract

Aging is accompanied by a decline in cognitive functions, including spatial memory, yet significant variability exists in the learning abilities of older individuals. Using a large cohort of aged and young male mice, we employed spatial discrimination testing in an 8-arm radial maze to investigate age-related differences in performance in spatial learning and to categorize individual memory phenotypes within the aged population. Despite a general learning ability across groups, aged mice showed slower acquisition rates compared to young counterparts, highlighting age-related cognitive difficulties in establishing or discriminating spatial representations. By modeling individual learning curves, we classified aged mice into two subgroups—normal learners (NL) and slow learners (SL)—based on learning speed. SL mice demonstrated significantly delayed spatial memory acquisition compared to NL and young mice, suggesting pronounced heterogeneity in cognitive aging. This method provides a robust framework to explore the neurobiological underpinnings of learning deficits and may inform the development of targeted interventions to mitigate age-related memory decline.

## 1 Introduction

Aging is associated with a progressive decline in both physical and cognitive functions, even in healthy individuals. Memory performance is particularly affected by these age-related changes, becoming evident in daily functioning (Cohen et al., 2019). However, it is important to note that the different types of memory do not undergo the same degree of disruption due to aging (Nilsson, 2003; Richmond and Burnett, 2022; Tran et al., 2021). Working memory and spatial memory, in particular, exhibit pronounced deficits in healthy older adults compared to their younger counterparts (Castillo Escamilla et al., 2023; Fernandez-Baizan et al., 2020; Newman and Kaszniak, 2000; Reinoso Medina et al., 2025; Wilkniss et al., 1997). While there are mixed results regarding sex differences, a recent study pointed out that males may be more prone to age-related decline in spatial memory (Febo et al., 2020; Gazova et al., 2013). While spatial memory shows a steady age-related decline (Fernandez-Baizan et al., 2020; Gazova et al., 2013), individual differences within the older adult population exist (Castillo Escamilla et al., 2023; Reynolds et al., 2019; Zhong et al., 2017). This variability suggests that a subgroup of older adults may be more prone to cognitive decline, potentially due to a combination of genetic susceptibility and environmental factors.

Individual differences in spatial memory abilities are also well-documented among both aged mice and rats (Drapeau et al., 2007; Gallagher et al., 1993; Newman et al., 2017). For instance, in the water maze, some aged rats exhibit significant deficits in spatial reference memory, while others perform comparably to younger animals, displaying no noticeable impairment. This effect is sex-independent since both females and males show a similar distribution of individual differences (Koh et al., 2022). It is further essential to recognize that different memory systems demonstrate varying degrees of susceptibility to aging (Chong et al., 2023; Drapeau et al., 2007; Gazova et al., 2013; Ladyka-Wojcik et al., 2021) and the underlying correlates of cognitive decline may differ between normal cognitive aging, impaired cognitive aging, and young adults in both rodents (Gallagher et al., 1993) and humans (Reynolds et al., 2019).

Investigating individual differences provides a valuable framework for understanding the neurobiological substrates associated with learning and memory impairments during aging. Our study characterized the cognitive performance of a large cohort of aged male mice submitted to spatial discrimination training in an 8-arm radial maze, comparing their performance to that of young adults. Although aged mice were able to learn the location of baited arms in the maze, we identified two distinct subgroups based on their speed of learning: normal (speed comparable to that of young mice) and slow learners. This categorization highlights the variability in cognitive performance within the aged population and introduces a novel tool for identifying individual differences in cognitive abilities. By pinpointing the cognitive characteristics of these aged subgroups, the underlying neurobiological mechanisms that contribute to variations in memory performance and cognitive aging can be further investigated. This research may lead to targeted interventions to mitigate memory decline in aging populations.

## 2 Materials and equipment

### 2.1 Materials and reagents

- Food pellets (dustless precision pellets rodent, 20 mg, BioServ, NJ, USA).
- Male C57BL/6 J mice (*n* = 18 aged 4–6 months for the young group and *n* = 111 aged 21–22 months for the older group), were obtained from the Janvier breeding center (Le Genest-St-Isle, France). Mice are housed in collective cages, with five mice per cage, and are provided with food and water *ad libitum* in a climate-controlled animal facility (22–23°C) with a 12-h artificial light–dark cycle (7 a.m. to 7 p.m.). Mice should be isolated and handled daily for 3 min, one week prior to the experiment. After isolation, they undergo food restriction to reach 85–90% of their initial *ad libitum* weight, continuing until the end of pretraining and training. All experimental protocols are conducted during the light phase (7 a.m. to 7 p.m.) of the light–dark cycle. Experimental procedures complied with official European Guidelines for the care and use of laboratory animals (directive 2010/63/UE) and were approved by the ethical committee of the University of Bordeaux (protocol A50120159).
- Ethanol 30%.

### 2.2 Equipment

#### 2.2.1 8-arm radial maze

The maze was made of grey PVC and consisted of a central platform (30 cm in diameter) with eight identical arms (62 cm long, 12 cm wide) extending outward in a symmetrical fashion (between arm angle of 45°) (Imetronic, Marcheprime, France). Each arm entrance was equipped with an automatic sliding door, remotely controlled via software by the experimenter from an adjacent room. Rewards, consisting of small dry milk pellets (one single 20 mg pellet per baited arm), were placed at the distal end of the chosen arms. Additionally, fixed distal cues were positioned on the walls of the experimental room (Hadzibegovic et al., 2025; Kohler et al., 2022).

#### 2.2.2 Mouse videotracking

The camera should be positioned above the radial maze to enable tracking of the mouse. With the camera monitoring the mouse’s behavior, software can track the animal’s movements and count the number of entries into each arm. This reduces the need for manual recording, which can be time-consuming and prone to mistakes.

#### 2.2.3 Software and datasets

- Mouse Tracking Software (Poly, Imetronic, Marcheprime, France). This software tracks the movements of the mice as they enter and exit the maze arms. Custom programs are created for pretraining (habituation) and training phases.
- GraphPad Prism (10.0) for statistical analysis of the results.

## 3 Procedure

Mouse home cages should be placed in a designated room adjacent to the training area, allowing the animals a 15-min habituation period prior to the initiation of the testing protocol. Lighting in the room should be adjusted to a low intensity (~50 lux), with stable distal cues maintained consistently throughout the pretraining and training phases. The maze should be cleaned with 30% ethanol before and after each mouse to remove the smell that can affect the mouse’s exploration of the maze, and it should be dried well to eliminate any residual ethanol odor. Subsequently, the Imetronic software should be launched, and the required protocol should be loaded. Start the camera to enable the software to track the mouse.

### 3.1 Food restriction

Mice should be isolated and handled daily for 3 min, one week prior to the experiment. Isolation is necessary to accurately monitor food intake, as individual food quantities are adjusted according to each mouse’s body weight and weight loss during food restriction. This approach ensures that the mouse’s body weight remains within the 85–90% range of their initial *ad libitum* weight. After isolation, food restriction continues until the end of pretraining and training.

### 3.2 First pretraining/habituation

On the first day of pretraining/habituation, open the tracking software and load the pretraining program P1 which will raise all doors to prevent access to the arms of the maze. Place a pellet at the center and the end of each arm. Gently carry the mouse to the center of the maze platform. Arms will be automatically open after 30 sec to allow the mouse to freely explore the maze for 10 min, records the sequence or visited arms and the program encourages the mouse to visit all arms by closing the doors of visited arms. The session ends once the mouse has visited all arms, ate the pellets and returned to the platform. Clean the maze with ethanol and replace the pellets for the next animal. Repeat the operation for all animals. This session is designed to associate the radial maze with food rewards.

### 3.3 Second pretraining/habituation

On the second day, open the tracking software and load the pretraining program P2. Place a pellet at the end of each arm only. Gently carry the mouse to the platform and allow it to explore the maze for 10 min. In contrast to P1, arms will not close after being visited, and the mouse can revisit arms without receiving additional food pellets. This session is designed to teach the mouse that the food is not refilled during the trial. Clean the maze and replace the pellets for the next mouse. Repeat the process for all mice.

### 3.4 Spatial discrimination

Place a pellet at the end of three arms only, spaced at 45, 90, and 135 degrees (e.g., arms 1, 2, and 4, or arms 2, 3, and 5). Each mouse can have a different set of baited arms, with the condition that baited arms remain constant for a given mouse throughout training (Days 1–8). Gently place the animal in the center of the maze and start the trial with all eight doors opening simultaneously after 30 sec. After a visit to an arm, the doors close for 4 s to prevent the mouse from adopting a clockwise or counterclockwise motor strategy during arm exploration. Such a non-mnemonic strategy would minimize reliance on integrated spatial memory representations. Allow the mouse to explore the maze until it finds the three baits and returns to the central platform. Replace the pellets and allow a 1-min interval between trials. The arms must be clean between each trial to remove residual odors and prevent the animal from identifying those visited during previous trials. Each daily training session consists of six consecutive trials, repeated over a total of eight days of training, with same conditions maintained throughout each session. Return the mice in their home cages and to the animal housing room after the completion each of daily training session. Measure body weight and provide food, one hour after the transfer, in adjusted amounts to maintain appropriate body weight.

## 4 Data analysis

During the habituation period, the time spent in the maze can be compared to ensure that all groups of mice have similar levels of engagement and familiarity with the maze before the testing phase begins. This comparison can be important to rule out potential differences in exploratory behavior that could influence performance during the testing trials.

### 4.1 Recording errors

For each trial, assess performance by recording the total number of errors defined as all visits to non baited arms and repeated visits to previously visited baited arms. Calculate the number of errors per session by averaging the number of errors across the six trials performed that day. Repeat this process from Day 1 to Day 8.

### 4.2 Analyzing raw errors

Use GraphPad Prism (10.0) to perform a two-way ANOVA with group (young vs. aged) and day (Day 1 to Day 8) as factors. Results are considered significant if the 95% confidence interval (*p* < 0.05) is reached.

### 4.3 Normalizing data

Normalize each animal data so that the number of errors on Day 1 corresponds to 100%. Exclude animals with an average error percentage exceeding 62.5% during the last three days of training, which represents the percentage of errors an animal would do if it visited at least once each of the 8 arms. This threshold indicates that the animal may not have learned the task by the end of training and can be considered a non-learner. Nine aged and one young animal were excluded based on this criterion.

### 4.4 Calculation of learning rate

The aim is to determine the amount of training needed by each mouse to reach half of their performance level achieved on Day 8– i.e., the “half-life” of the curve. Non-linear exponential regression on GraphPad Prism is used to model individual learning curves, smoothing any irregularities in performance. To assess the goodness of fit for the non-linear exponential regression, we used the coefficient of determination (R^2^), which is appropriate for non-linear models. Select animals with a coefficient of determination greater than 0.75 for further analysis. A coefficient of determination of 0.75 means that 75% of the variation in the data is explained by the fitted model, reflecting a satisfactory, though not perfect, match. This suggests that the model is generally a good predictor of the observed trends. If a stricter threshold (e.g., *r* > 0.85) were used, more animals would be excluded from the analysis, potentially reducing statistical power and introducing bias. The learning speed is represented by the “half-life” of the curve.

### 4.5 Group comparison

Perform a Shapiro–Wilk test to assess the normal distribution of the data. If the values are normally distributed, use a t-test to compare the learning speed between the young and aged groups. If not, use the non-parametric Mann–Whitney (M-W) test.

### 4.6 Revealing slow vs. normal aged learners

Divide the aged group into two based on the median to create “normal speed” and “slow speed” subgroups. Compare learning speed across the three groups (young, normal speed aged, slow speed aged) using a two-way ANOVA if the distribution is normal. If not, use the Kruskal-Wallis (K-W) test.

### 4.7 Distinguishing spatial reference and working memory errors during spatial discrimination learning

Solving the spatial discrimination task requires both spatial reference memory and working memory. These two memory forms can be apprehended by scoring specific error types. A reference memory error was defined as the first visit to a non-baited arm within a trial, with a maximum of five errors per trial. Working memory errors were identified as repeated visits to the same arm during a trial. However, since reference memory enables the avoidance of non-baited arms, only re-entries into baited arms were considered true working memory errors. Thus, repeated visits to baited arms within a trial were scored as working memory errors.

## 5 Results

### 5.1 Age-related changes in spatial learning and memory in the 8-arm radial maze

**Figure 1:**
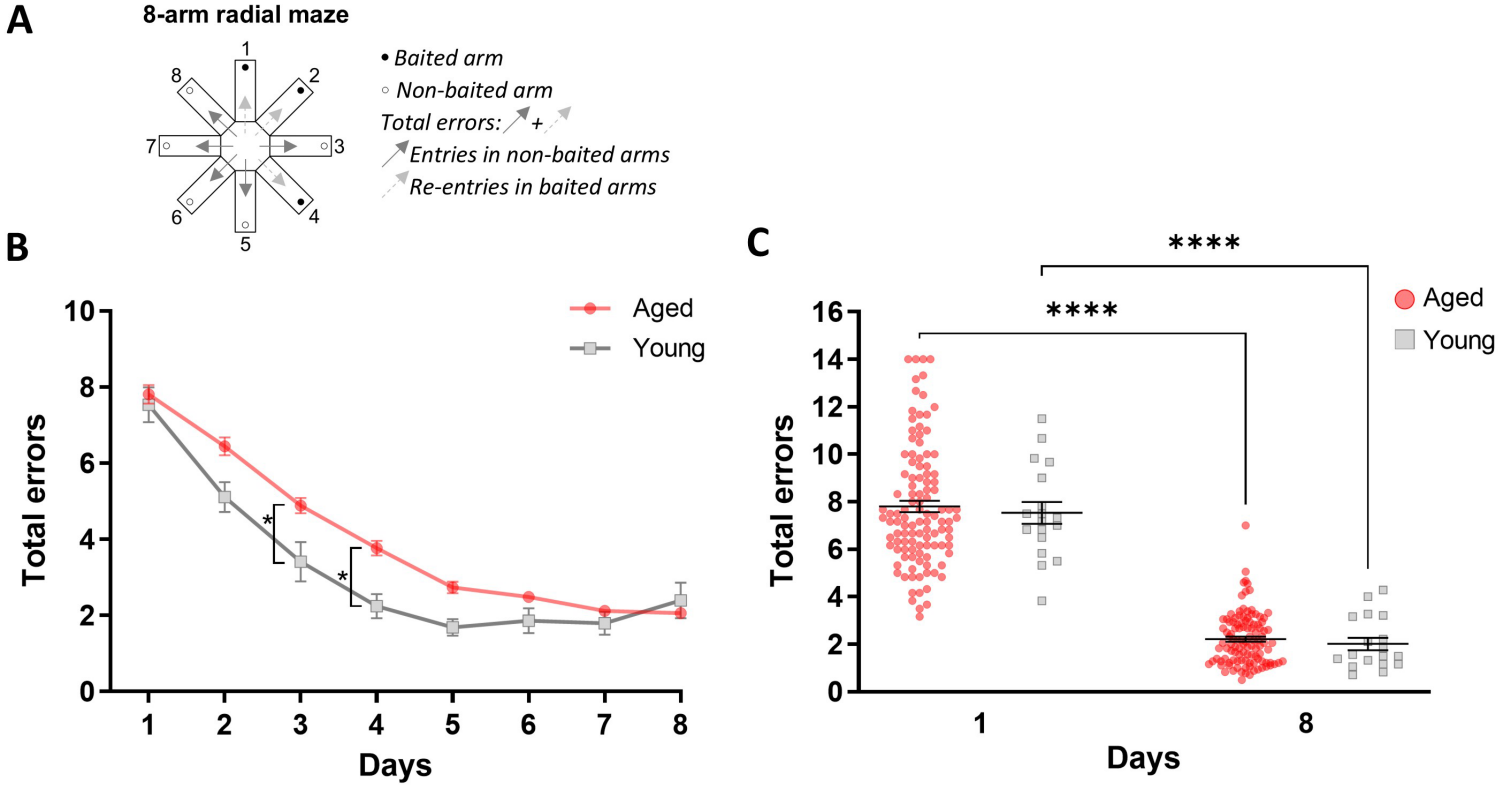
Age-related differences in spatial discrimination learning in the 8-arm radial maze. **(A)** Schematic illustration of the 8-arm radial maze highlighting baited and non-baited arms, as well as the total number of errors scored when an animal enters non-baited arms and re-enters a previously visited baited arm. **(B)** Memory performance is expressed as the mean total number of errors (±SEM) over six daily trials during the eight training days. **(C)** Total errors (±SEM) during the first and last training sessions. Statistical analysis: *p < 0.05, ****p < 0.0001, *n* = 18 to 111.

During the second day of habituation (P2), young and aged mice spent a similar amount of time in the maze (570.3 s ± 116.9 versus 575.6 s ± 42.44 respectively; M-W, U = 282, *p* = 0.25). Further, both young and aged mice managed to learn the positions of the baited arms over 8 days of training. However, a two-way ANOVA revealed an effect of age [*F*_(1,127)_ = 5.99; *p* = 0.016] and testing days [*F*_(7,889)_ = 89.71; *p* < 0.0001] on learning performance. There was a significant interaction between age and days [*F*_(7,889)_ = 2.73; *p* = 0.019], indicating that young mice acquired the correct arm choices faster than aged mice. While both groups improved their performance over time, young mice demonstrated quicker adaptation to the task, with notable differences on specific days (day 3 and day 4; Figures 1A,B). The total number of errors committed on the first and last day of training did not differ between the groups [*F*_(1,127)_ = 0.13; *p* = 0.72; Figure 1C], suggesting that both groups started with a similar baseline of errors and that no confounding factors, such that a performance effect in aged mice, influenced learning progression. This absence of a difference in errors on the last day of testing further supports the conclusion that both young and aged mice successfully learned the task, although younger mice had a faster spatial memory acquisition rate than older mice, highlighting age-related differences in processing spatial memory representations.

### 5.2 Modeling learning speed using nonlinear exponential regression to assess age-related differences in spatial learning

**Figure 2:**
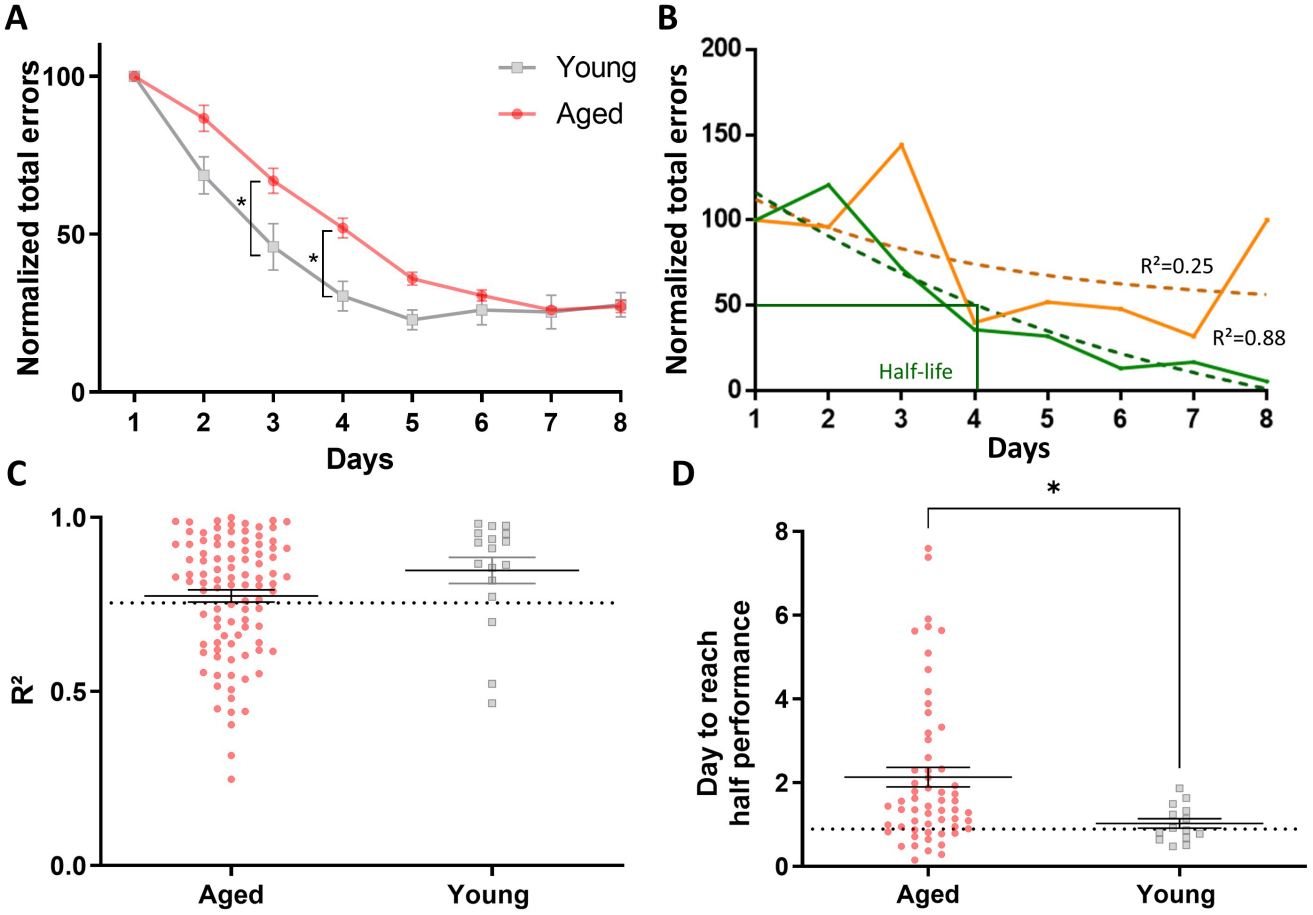
Modeling learning speed using nonlinear exponential regression to assess age-related differences in spatial learning. **(A)** Normalized learning curves for groups of aged and young mice during the spatial learning task in the 8-arm radial maze. Data for each animal were normalized to 100% on the first day (±SEM). Statistical analysis: *p < 0.05, *n* = 17 to 102. **(B)** Examples of individual learning curves for two mice, one in orange and the other in green. The solid lines represent their actual learning curves, making the learning rate difficult to calculate due to performance variations across days. Therefore, we modeled a learning curve for each mouse using the nonlinear exponential regression method. A fit test was performed to ensure that the modeled curves (dotted lines) accurately represent the actual learning curves. Half-life represents the number of days corresponding to 50% of the normalized total number of errors. **(C)** Representation of the R^2^ values for each individual in the groups of aged and young mice. Based on the selection criterion (R^2^ > 0.75), only the “green mouse” in **(B)** met the criterion and was selected to determine its learning rate. **(D)** Learning speed as a function of mouse age (±SEM), measured by the number of sessions required to reach the half performance criterion. Aged mice took longer to reach this criterion, indicating a slower learning speed than young mice. The dotted line presents the median performance of young animals. Statistical analysis: *p < 0.05, *n* = 14 to 59.

We further examined individual mouse learning curves after normalizing the data by setting the number of errors on day 1 as 100% of the errors made (Figure 2A). These adjustments smooth the reduction of interindividual differences while making learning more salient, facilitating the discrimination of individuals who may not have learned the task by the end of the training. Two representative learning curves, one shown in orange and the other in green, demonstrate how learning curves could vary in the population (Figure 2B). The solid lines represent the actual learning curves, which exhibited considerable variation across sessions, complicating the calculation of a precise learning rate. To address this, we applied a nonlinear exponential regression method to model the learning curve for each mouse (Figure 2A). A coefficient of determination R^2^ test was calculated to verify that the modeled curves accurately represented the actual learning data. The R^2^ values for each individual in both the young and aged groups are shown in Figure 2C. Based on a selection criterion (R^2^ > 0.75), only mice above the threshold (Figure 2C) were selected for further analyses. In the example in Figure 2B, only the mouse represented by the green lines met this threshold and was selected for further analysis to determine its learning rate. Out of the 111 aged animals, 9 failed to learn the task, and 34 (representing 33.3% of the animals) were excluded from the analysis due to an R^2^ value below the chosen threshold. In contrast, only 1 young animal out of 18 failed to learn the task, and 3 (representing 17.4% of the animals) were excluded for not meeting our R^2^ criteria. The learning speed, measured for each mouse by the number of days to reach half of its performance level achieved on Day 8, is shown in Figure 2D as a function of age. A Mann–Whitney test indicated that aged mice took significantly longer to reach this criterion compared to young mice (M-W, U = 236, *p* = 0.012), supporting the conclusion that aging affects the rate of spatial learning (Figure 2D).

### 5.3 Learning speed in subgroups of aged mice reveals slow and normal learners

**Figure 3:**
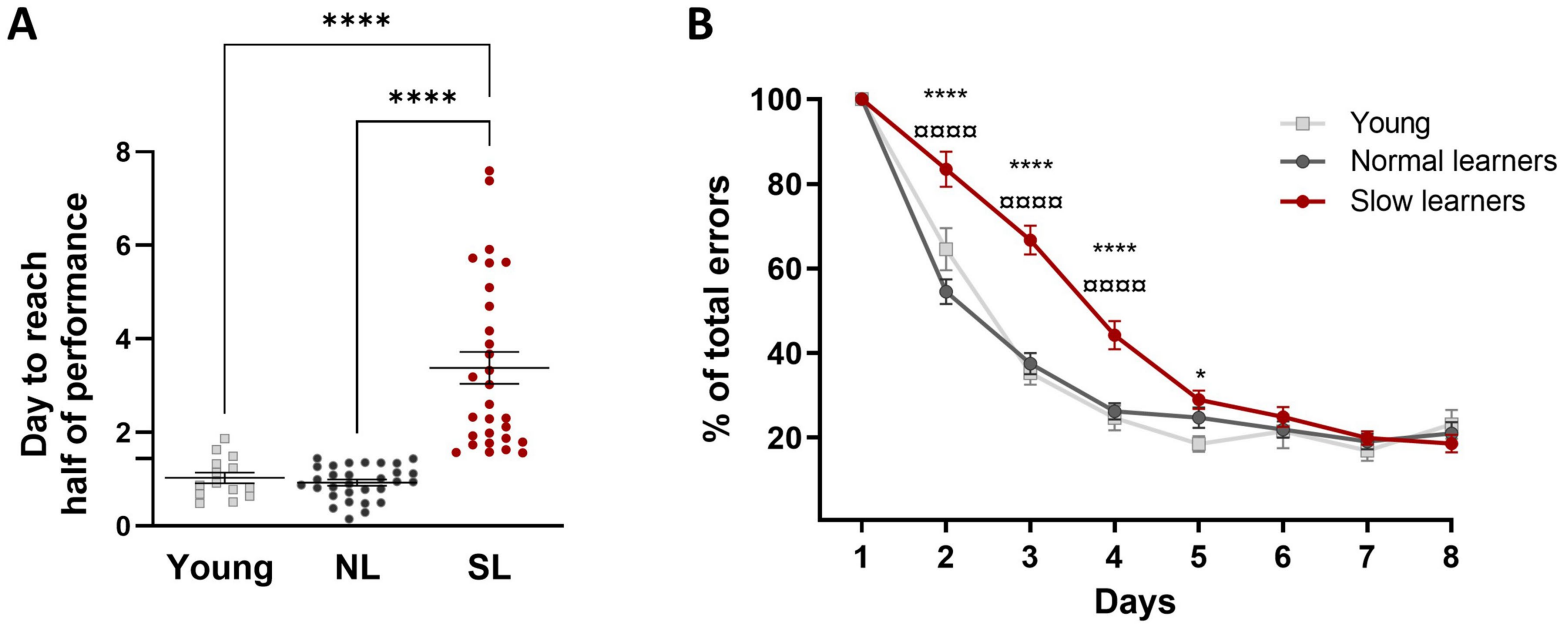
Learning speed in subgroups of aged mice distinguishing slow learners (SL) from normal learners (NL). **(A)** Aged mice in the SL group took longer to reach the half performance criterion, indicating a slower learning speed compared to young mice. There was no difference between the young and NL groups. Statistical analysis: ****p < 0.0001, *n* = 14 to 30. **(B)** Normalized learning curves for NL and SL aged mice and young mice during the spatial learning task in the 8-arm radial maze. Data for each animal were normalized to 100% on the first day (±SEM). Statistical analysis: ****p < 0.0001, *p < 0.05, versus young mice; ^¤¤¤¤^*p* < 0.0001 versus NL mice; *n* = 14 to 30.

We identified slow learners (SL) and normal learners (NL) within the aged group based on the median performance of the young group. Specifically, aged mice that reached 50% performance below the median of the young group’s performance were classified as NL, while those that reached 50% performance above the median were classified as SL. A Kruskal-Wallis test revealed that SL mice took significantly longer to reach the 50% performance criterion compared to young mice and NL (K-W, χ^2^ = 50.13, *p* < 0.0001, Figure 3A), indicating a slower learning speed in this group. There was no significant difference between the young and NL groups. Further, the analysis of normalized learning curves for NL and SL aged mice and young mice with a two-way ANOVA revealed that SL mice made more errors compared to both young and NL mice [*F*_(2,70)_ = 24.97; *p* < 0.0001; Figure 3B]. This difference in error rates is not due to differences in maze exploration, as no significant differences in time spent exploring the maze were observed between young, NL and SL groups during the second habituation period (570.3 s ± 116.9 versus 576.3 s ± 55.56 versus 575.1 s ± 63.21 respectively; K-W, χ^2^ = 1.71, *p* = 0.42). In addition, a Chi-square analysis revealed no significant differences in the distribution of testing for NL and SL across three time periods: morning (7 a.m. to 11 a.m.), midday (11 a.m. to 3 p.m.), and afternoon (3 p.m. to 7 p.m.; Supplementary Figure 1) for days with either no difference (Day 1 and Day 8; Chi-square, Day 1: χ^2^ (2, *N* = 59) = 0.04, *p* = 0.98; Day 8: χ^2^ (2, *N* = 59) = 0.98, *p* = 0.61) or a significant difference in memory performance (Day 4: χ^2^ (2, *N* = 59) = 0.14, *p* = 0.93). These results suggest that exploration and time of day when the mice were tested do not explain the observed differences in the speed of learning between NL and SL.

### 5.4 Spatial reference and working memory performance in aged normal and slow learners

Our training procedure in the 8-arm radial maze enables us to identify two main types of errors, namely reference and working memory errors, which are not underlined by the same memory systems. We first examined working memory errors (Supplementary Figures 2A–C). While young and aged mice progressed over training days (two-way ANOVA, main effect of days: *F*_(5.204, 385.1)_ = 42.29, *p* < 0.0001), there was a significant group effect [*F*_(2, 74)_ = 4.47, *p* < 0.01] with a significant group x days interaction [*F*_(14, 518)_ = 3.95; *p* < 0.0001], indicating that aged mice from both the NL and SL subgroups were slower in avoiding repeated visits into baited arms (Supplementary Figure 2B). Accordingly, a higher number of cumulative working memory errors was observed in NL (Tukey’s multiple comparison test: *p* = 0.0002) and SL subgroups of aged mice (Tukey’s multiple comparison test: *p* = 0.0018) compared to young mice (Supplementary Figure 2C). Also, numbers of working memory errors committed by NL and SL aged mice were similar, as revealed by a two-way ANOVA [*F*_(1, 57)_ = 0.098, *p* = 0.76; Supplementary Figure 2B]. Numbers of cumulative working memory errors on Day 4 for NL and SL subgroups were also comparable (Tukey’s multiple comparison test: *p* = 0.79; Supplementary Figure 2C). The performance of all groups was similar on Day 1, thus ruling out a performance effect due to aging (Post-hoc Šídák’s multiple comparisons test: NL versus SL: *t*_(54.87)_ = 2.59, *p* = 0.10; NL versus Y: *t*_(39.26)_ = 2.59, *p* = 0.27; SL versus Y: *t*_(32.91)_ = 0.11, *p* > 0.99).

We next examined the progression of reference memory errors made by young mice and NL and SL subgroups of aged mice (Supplementary Figures 2D–F). While young and aged mice progressed over training days (two-way ANOVA, main effect of days: *F*_(4.711, 348.6)_ = 155, *p* < 0.0001), there was no significant group effect [*F*_(2, 74)_ = 2.091, *p* = 0.13] with a significant group x days interaction [*F*_(14, 518)_ = 2.45, *p* = 0.0024]. Close examination of the progression of reference memory errors made by young mice revealed a non-linear pattern. Their performance started to plateau after 4 days of training and stabilized thereafter (Supplementary Figure 2E). Post-hoc analyses indeed revealed that the number of reference memory errors on Day 4 was significantly lower than on Day 3 (Sidak’s multiple comparison test: *p* = 0.013) and not different from Day 5 (Sidak’s multiple comparison test: *p* = 0.97). In contrast to NL mice, SL mice needed more training days to reach a level of task mastery comparable to that of young mice. Thus, the cumulated number of reference memory errors after 4 training days was higher in SL mice than in young mice (Tukey’s multiple comparisons test: *p* = 0.011) or in NL mice (Tukey’s multiple comparisons test: *p* = 0.014; Supplementary Figure 2F). Overall, the number of reference memory errors appears to discriminate between SL and NL subgroups of aged mice, suggesting that reference memory is more resistant to aging, contrary to working memory.

Last, since each daily training session consisted of six trials, intra-session progression for young and SL and NL mice was also assessed by averaging and comparing the total number of errors on the first three and last three trials of the day (Supplementary Figure 3). The results revealed only slight variations between aged SL and NL mice when compared to young animals (two-way ANOVA, group effect: *F*_(2, 1,184)_ = 7.88, *p* = 0.0004). However, these variations are not sufficient to account for the performance differences observed between SL and NL subgroups over training days, suggesting that aging-induced slower learning is likely due to a combination of reduced intra-and inter-session progression.

## 6 Discussion

In line with cognitive studies in aged humans, we observed significant variability among aged mice submitted to spatial discrimination testing in the 8-arm radial maze. By analyzing the performance of a large cohort of aged mice, we developed a novel method based on statistical criteria to quantify and categorize memory deficits observed during spatial learning. Our analysis revealed that mice, regardless of age, were ultimately able to complete the task successfully, with no significant differences between young and aged mice by the final learning session. However, a closer examination highlighted that aging primarily slows the speed of learning rather than impairing the overall ability to learn (Barnes and McNaughton, 1985).

These findings differ from previous studies using other spatial learning paradigms (Drapeau et al., 2007; Gage et al., 1988; Haberman et al., 2017; Lund et al., 2004). In some of these earlier studies, the limited number of learning sessions may have prevented aged animals from fully demonstrating their learning capabilities. To better capture the nuances in learning speed, we developed a method that measures the amount of training required for animals to reach half of their performance level achieved upon training completion. This approach, grounded in nonlinear regression modeling, accounts for variability in performance, such as occasional errors in individual sessions. Using this method, we identified two distinct populations within the aged cohort: one group with learning speeds comparable to young mice (normal-speed learners) and another group with significantly slower learning speeds (slow learners). This reliable categorization tool provides a valuable framework for examining aging-related interindividual differences and offers potential for further unraveling the neurofunctional mechanisms underlying these differences. A similar approach could be applied to a larger cohort of young animals to determine whether the same observations emerge in a non-aged population. This would help exclude the possibility that the identified learning subgroups may not be specific to aging but rather reflect inherent variability in learning abilities.

While variables such as visual acuity, motor function, or anxiety can influence performance in maze-based tasks, our results do not indicate that these factors systematically differed across groups. Notably, all groups began with comparable levels of memory errors on Day 1 and showed converging performance by Day 8. Furthermore, time spent in habituation, which could be sensitive to both anxiety and motor deficits, did not differ significantly between groups. These findings support the interpretation that the differences in learning trajectories, particularly between the slow and normal learner groups, primarily reflect variations in cognitive processing rather than non-cognitive impairments.

Because aging has been shown to influence circadian rhythms (Kondratova and Kondratov, 2012) which in turn can affect spatial memory performance (Morales-Delgado et al., 2018; Smarr et al., 2014; Van Drunen and Eckel-Mahan, 2023; Winocur and Hasher, 2004), future studies specifically interested in the potential effects of circadian rhythm could investigate its impact on memory performance in aged animals.

It is also possible to interpret the variability in learning speed observed among aged mice by differences in their strategies during spatial learning. Studies using the Morris water maze have shown that animals can adopt specific strategies to compensate for memory deficits. For instance, aged animals often perform comparably to younger ones when employing egocentric strategies (relying on their own orientation) but show pronounced deficits when required to use allocentric strategies [relying on external spatial cues—(Gage et al., 1988; Lund et al., 2004)]. Similar trends are observed in humans, where strategic choices differ between young and older individuals and are closely linked to memory performance (Ariel et al., 2015; Burger et al., 2017). Although our experimental approach cannot entirely rule out the possibility that aged slow and normal learners employ different strategies, we minimized the use of egocentric strategies by closing the doors of the arms for four seconds between each arm visit. This intervention not only reduces the animals’ opportunity to rely on egocentric strategies but also encourages them to adopt an allocentric approach. Instead, they may have used allocentric strategies, which depend on spatial cues, and rely on an intact hippocampal network, while egocentric strategies are more dependent on the striatal network (Colombo et al., 2003; Packard and McGaugh, 1996). The hippocampus, a critical structure for spatial information processing, is particularly sensitive to aging and undergoes numerous structural and functional changes (Rosenzweig and Barnes, 2003; Shivarama Shetty and Sajikumar, 2017) that may have been more prominent in aged mice of the SL subgroup.

Our novel statistical method offers an opportunity to refine future behavioral and biochemical studies on aged individuals. Previous studies have identified various biomarkers that differ between young and aged individuals by studying cognitive decline, particularly in spatial memory (Febo et al., 2020; Foster, 2012; Jabès et al., 2021; Myrum et al., 2022). By applying our method, researchers can more precisely track the evolution of these biomarkers as individuals age, especially in the hippocampus, a critical structure for spatial tasks. Future studies integrating behavioral analyses with biological markers could shed light on how aging-related molecular changes correlate with learning efficiency and strategic choices. Such research would provide a more comprehensive understanding of cognitive aging, paving the way for targeted interventions to mitigate age-related memory decline.

## Data Availability

The raw data supporting the conclusions of this article will be made available by the authors, upon reasonable request.

## Supplementary material

SUPPLEMENTARY FIGURE 1 Distribution of aged normal and slow learners tested at different times (morning, midday and afternoon) during the day. No significant differences were observed between normal and slow learners for three time periods: morning (7 a.m. to 11 a.m.), midday (11 a.m. to 3 p.m.), and afternoon (3 p.m. to 7 p.m.), indicating that testing time was not a confounding variable in our experiments. Bar graphs display the percentage of animals tested in each time window across D1, D4 and D8 training days.

SUPPLEMENTARY FIGURE 2 Differentiation between normal learners (NL) and slow learners (SL) in working and reference memory errors. **(A)** Schematic illustration of the 8-arm radial maze, highlighting baited and non-baited arms, as well as the working memory errors that occur when an animal re-enters a previously visited baited arm. **(B)** Mean working memory errors (± SEM) across six daily trials during the eight training days. **(C)** Mean cumulative working memory errors over the first four training days (D1 to D4) with six daily sessions per mouse. Both SL and NL mice exhibit significantly more working memory errors compared to young mice. **(D)** Schematic illustration of the maze showing the reference memory errors that occur when an animal makes a first entry in a non-baited arm. **(E)** Mean reference memory errors (± SEM) over six daily trials during the eight training sessions. **(F)** Mean cumulative reference memory errors over the first four training days (D1 to D4) with six daily sessions per mouse. SL mice show significantly more reference memory errors compared to both NL and young mice, suggesting a preservation of cognitive performance through the retention of reference memory in NL. Statistical analysis: **p* < 0.05, ***p* < 0.01, ****p* < 0.001; *n* = 14 to 30.

SUPPLEMENTARY FIGURE 3 Age-related differences in the intra-session learning measured over the six daily trials during the eight training days. Mean total errors (±SEM) of the first vs. last three trials of the day, revealing only a slight variation between SL and NL compared to young mice. Statistical analysis: **p* < 0.05, *n* = 14 to 30.
