# Supplementary figures and images for: Normal and slow learners: a new discriminative method based on the speed of spatial learning in aged mice
Source: Front Aging Neurosci. 2025 Jun 6;17:1567929. doi: 10.3389/fnagi.2025.1567929 (PMC12179159; doi:10.3389/fnagi.2025.1567929)

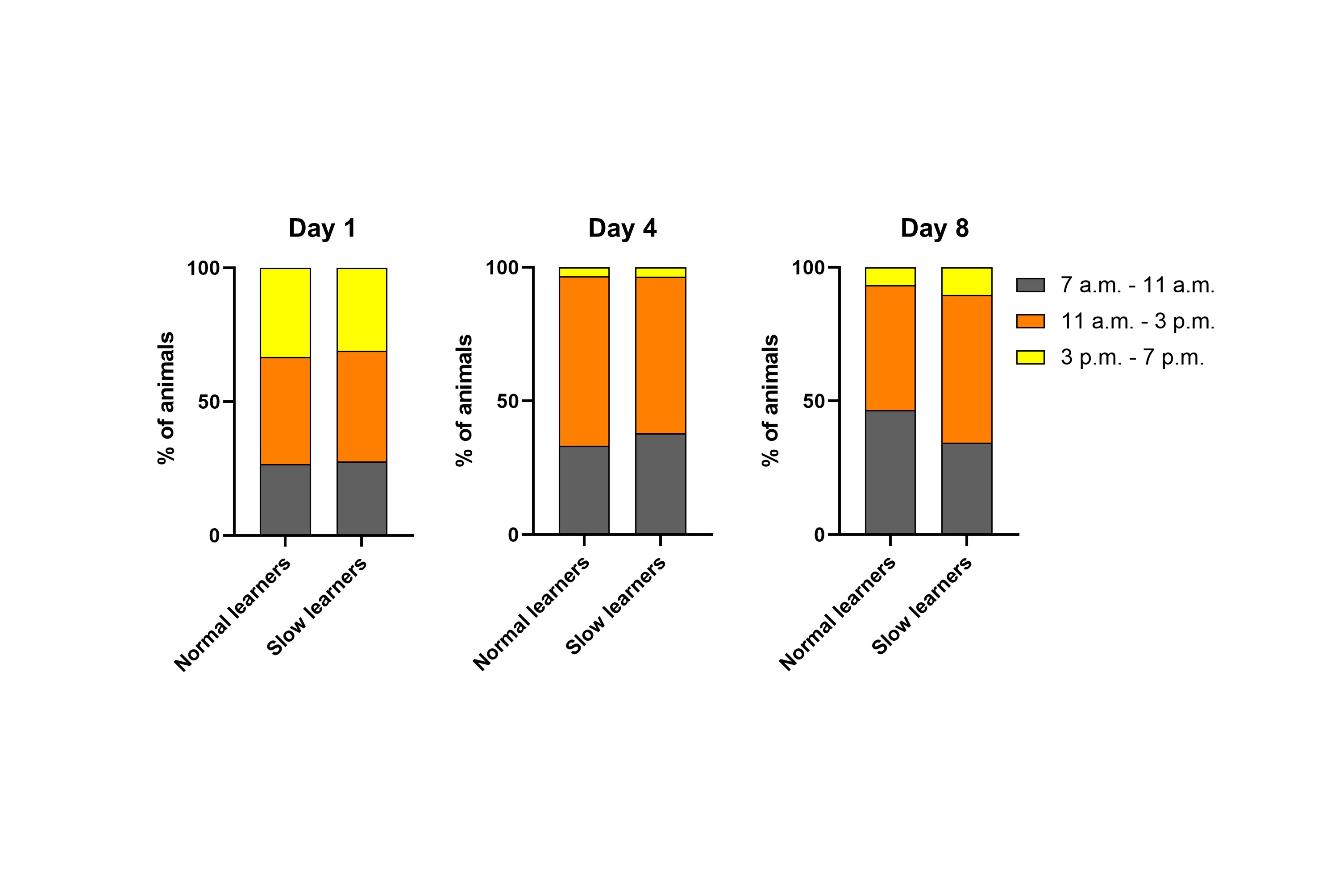

Supplement: SUPPLEMENTARY FIGURE 1 — Distribution of aged normal and slow learners tested at different times (morning, midday and afternoon) during the day. No significant differences were observed between normal and slow learners for three time periods: morning (7 a.m. to 11 a.m.), midday (11 a.m. to 3 p.m.), and afternoon (3 p.m. to 7 p.m.), indicating that testing time was not a confounding variable in our experiments. Bar graphs display the percentage of animals tested in each time window across D1, D4 and D8 training days. [file Image_1.jpeg]

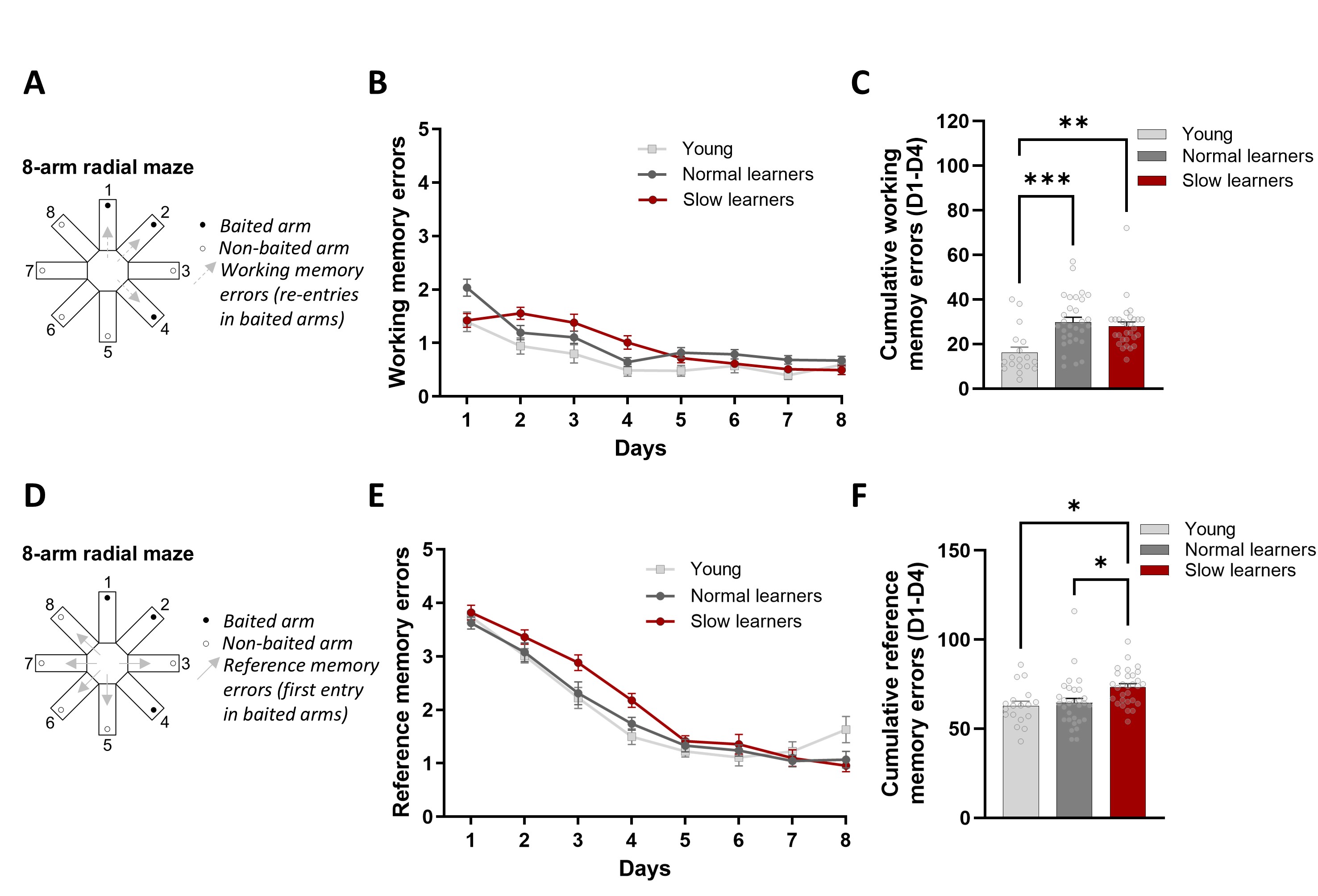

Supplement: SUPPLEMENTARY FIGURE 2 — Differentiation between normal learners (NL) and slow learners (SL) in working and reference memory errors. (A) Schematic illustration of the 8-arm radial maze, highlighting baited and non-baited arms, as well as the working memory errors that occur when an animal re-enters a previously visited baited arm. (B) Mean working memory errors (± SEM) across six daily trials during the eight training days. (C) Mean cumulative working memory errors over the first four training days (D1 to D4) with six daily sessions per mouse. Both SL and NL mice exhibit significantly more working memory errors compared to young mice. (D) Schematic illustration of the maze showing the reference memory errors that occur when an animal makes a first entry in a non-baited arm. (E) Mean reference memory errors (± SEM) over six daily trials during the eight training sessions. (F) Mean cumulative reference memory errors over the first four training days (D1 to D4) with six daily sessions per mouse. SL mice show significantly more reference memory errors compared to both NL and young mice, suggesting a preservation of cognitive performance through the retention of reference memory in NL. Statistical analysis: *p < 0.05, **p < 0.01, ***p < 0.001; n = 14 to 30. [file Image_2.jpeg]

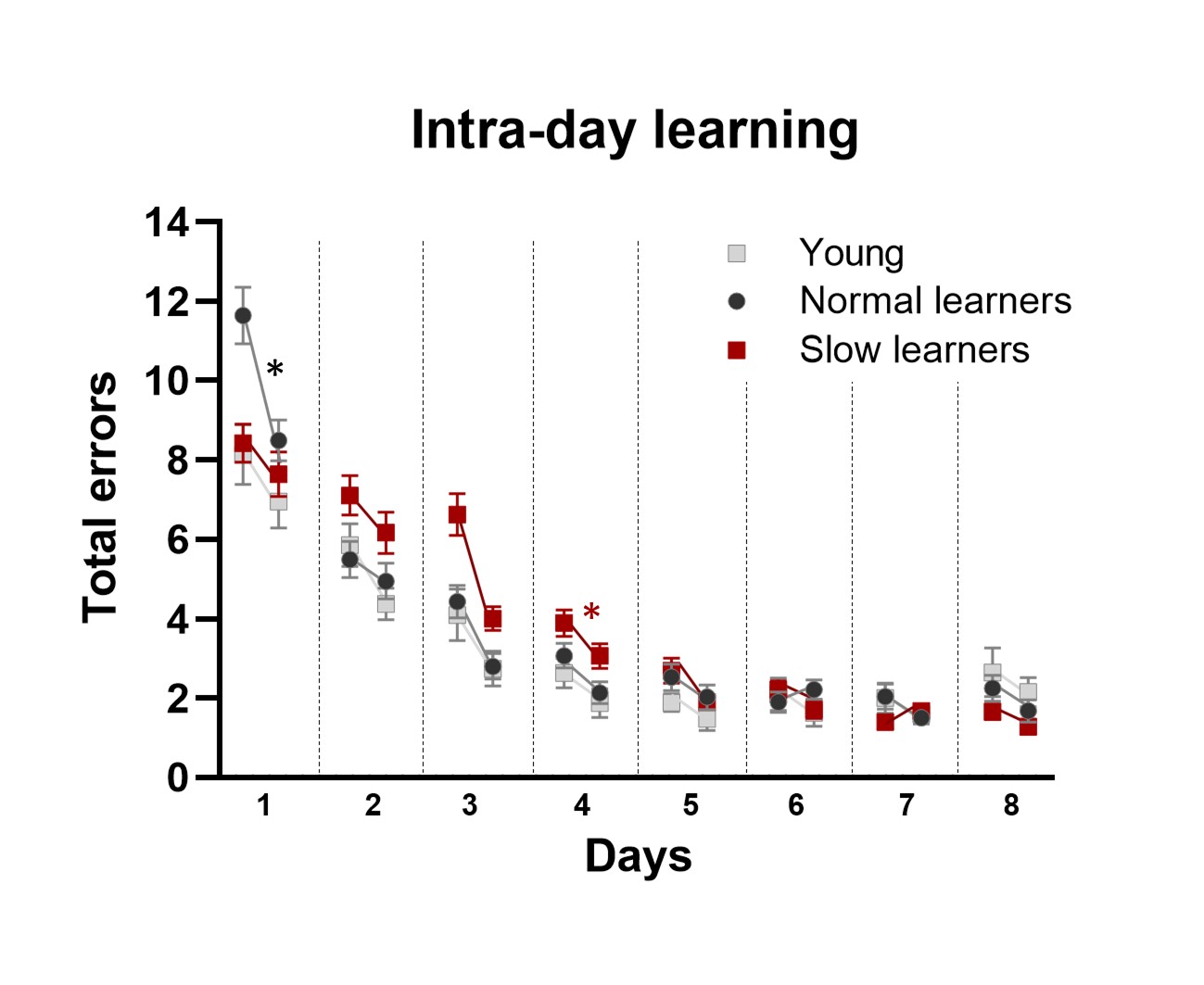

Supplement: SUPPLEMENTARY FIGURE 3 — Age-related differences in the intra-session learning measured over the six daily trials during the eight training days. Mean total errors (±SEM) of the first vs. last three trials of the day, revealing only a slight variation between SL and NL compared to young mice. Statistical analysis: *p < 0.05, n = 14 to 30. [file Image_3.jpg]
